# Einsatz chirurgischer Simulatoren in der Weiterbildung – eine deutschlandweite Analyse

**DOI:** 10.1007/s00104-020-01332-2

**Published:** 2021-01-05

**Authors:** Stefanie Brunner, Juliane Kröplin, Hans-Joachim Meyer, Thomas Schmitz‑Rixen, Tobias Fritz

**Affiliations:** 1 Klinik und Poliklinik für Allgemein‑, Viszeral‑, Tumor- und Transplantationschirurgie, Universitätsklinikum Köln, Kerpener Str. 62, 50937 Köln, Deutschland; 2 Klinik für Mund‑, Kiefer- und Gesichtschirurgie – Plastische Operationen, Helios Kliniken Schwerin, Wismarsche Str. 393–397, 19055 Schwerin, Deutschland; 3 Deutsche Gesellschaft für Chirurgie, Berlin, Deutschland; 4 Berufsverband der Deutschen Chirurgen e. V. (BDC), Berlin, Deutschland; 5 Klinik für Gefäß- und Endovascularchirurgie, Universitätsklinikum Frankfurt, Frankfurt am Main, Deutschland; 6 Klinik für Unfall‑, Hand- und Wiederherstellungschirurgie, Universitätsklinikum des Saarlandes, Homburg, Deutschland; 7 Perspektivforum Junge Chirurgie, Deutsche Gesellschaft für Chirurgie, Berlin, Deutschland

**Keywords:** Simulator, Chirurgie, Weiterbildung, Perspektivforum, Deutsche Gesellschaft für Chirurgie, Simulator, Surgery, Further education, Perspective forum, German Society of Surgery

## Abstract

**Hintergrund:**

Die chirurgische Facharztweiterbildung erfordert neben dem Erlernen theoretischen Wissens ebenfalls den Erwerb praktisch-chirurgischer Kompetenzen. Eine Alternative zur Aus- und Weiterbildung am Patienten stellen simulationsbasierte Lehrkonzepte dar. Ziel der vorliegenden Studie ist die Analyse der Verteilung und des Einsatzes chirurgischer Simulatoren in deutschen Kliniken.

**Methoden:**

Die Datenanalyse erfolgte auf Basis eines individuellen Onlinefragebogens mit insgesamt 19 standardisierten Fragen. Dieser wurde über die E‑Mail-Verteiler der deutschen chirurgischen Fachgesellschaften an die leitenden chirurgischen Klinikärzte versendet.

**Ergebnisse:**

Insgesamt 267 vollständige Antwortdatensätze wurden analysiert (Rücklaufquote 12,0 %). 84,0 % der Teilnehmer gaben ihre Tätigkeit an einem Lehrkrankenhaus an. Zum Zeitpunkt der Untersuchung waren 143 chirurgische Simulatoren an 35,0 % der in die Auswertung eingeschlossenen Kliniken vorhanden. Regional zeigten sich deutliche Unterschiede zwischen den einzelnen Bundesländern. 21,1 % der Teilnehmer, an deren Klinik kein Simulator zur Verfügung steht, planten eine Neubeschaffung. Studierende (41,1 %) und Ärzte in Weiterbildung (ÄiW, 32,5 %) nutzten das Simulationstraining am häufigsten. Eine Integration in die chirurgische Weiterbildung bestand zu 81,8 % nicht. 94,0 % der beteiligten Kliniken zeigten Interesse an einer zukünftigen Integration in die chirurgische Facharztweiterbildung.

**Schlussfolgerung:**

Die vorliegenden Ergebnisse bestätigen die besondere Bedeutung des simulationsbasierten Trainings für die chirurgische Weiterbildung an deutschen Kliniken. Gleichzeitig bestehen deutliche Informationsdefizite über das Nutzungsverhalten sowie eine defizitär empfundene Integration des Simulationstrainings in die chirurgische Weiterbildung.

## Abstract (en)

**Background:**

In addition to learning theoretical knowledge, the medical specialist training in surgery necessitates the acquisition of practical surgical competences. Simulation-based teaching concepts represent an alternative to education and advanced training on patients. The aim of this study was to analyze the distribution and implementation of surgical simulators in German hospitals.

**Methods:**

The data analysis was carried out based on an individual on-line questionnaire with a total of 19 standardized questions. This was sent to the senior surgeons in hospitals and clinics via the email distributors of specialist societies for surgery in Germany.

**Results:**

A total of 267 complete datasets were analyzed (response rate 12%). Of the participants 84% reported that they were active in a teaching hospital. At the time of the investigation 143 surgical simulators were in use at 35% of the hospitals and clinics included in the evaluation. There were clear regional differences between the individual federal states. Of the participants, 21.1% did not have a simulator at the hospital but the acquisition of one was planned. Simulation training was most frequently used by students (41.1%) and physicians during further education (32.5%). Simulators were not integrated into advanced surgical training in 81.8%. Of the participating hospitals, 94% showed an interest in integration into surgical specialist training in the future.

**Conclusion:**

The results of this survey confirmed the special importance of simulation-based training for surgical education in German hospitals; however, at the same time there were clear deficits in information concerning user behavior and a deficiency in the perceived integration of simulation training in advanced training for surgery.

## Hintergrund

Die technischen Innovationen und der wissenschaftliche Fortschritt in der Medizin stellen sowohl den chirurgischen Nachwuchs als auch die Weiterbilder vor immer neue Herausforderungen [1]. Neben dem Erlernen eines umfangreichen Portfolios allgemeiner und chirurgischer Krankheitsbilder gewinnen zunehmend interpersonelle Faktoren und der ökonomische Umgang mit begrenzten Ressourcen an Bedeutung. Gleichzeitig wächst von der jungen Ärztegeneration die Forderung nach guten Arbeitsbedingungen und einer strukturierten Weiterbildung [2]. Insbesondere die chirurgischen Fächer stehen vor der Herausforderung trotz des zunehmenden Dokumentationsaufwands und der Regulation durch das Arbeitszeitgesetz den praktischen und theoretischen Kompetenzerwerb in der Weiterbildung zu gewährleisten. Für das Erlernen operativer Techniken – einschließlich roboterunterstützter Eingriffe – sollten zukünftige Weiterbildungskonzepte sowohl eine hohe Patientensicherheit als auch eine exzellente Qualität der Weiterbildung gewährleisten. Dies offeriert, unter Berücksichtigung geeigneten Motivationsfaktoren für die Wahl einer chirurgischen Fachdisziplin, ebenso die Möglichkeit, dem zunehmenden Mangel an chirurgischen Fachkräften entgegenzuwirken [3, 4]. Um diesen Anforderungen gerecht zu werden, wurden und werden weltweit unterschiedliche Konzepte entwickelt [5].

Das deutsche Weiterbildungssystem beruht – auch im Zuge der novellierten Weiterbildungsordnung mit dem Ziel einer kompetenzbasierten Weiterbildung – weiterhin überwiegend auf Fallzahlen [6]. Umfragestudien haben gezeigt, dass die Weiterbildung in Deutschland zum Teil als unstrukturiert empfunden wird [2]. Dennoch bieten wenig strukturierte Kurrikula auch Vorteile, z. B. durch eine flexible Anpassung an den Klinikbetrieb sowie an die individuellen Anforderungen der Weiterzubildenden. Zu berücksichtigen ist ebenfalls das bestehende Abhängigkeitsverhältnis zwischen Weiterbildungsbefugten und Ärzten in Weiterbildung (ÄiW), da die operativen Eingriffe durch den Weiterbildungsbefugten zugeteilt werden [7].

Im internationalen Vergleich variieren die Anforderungen an die Weiterbildung und die Struktur derselben zum Teil ebenfalls stark. So streben einige Länder, wie z. B. Kanada oder die Schweiz, ein streng strukturiertes Kurrikulum mit Zwischenprüfungen an. Unterschiede bestehen hierbei sowohl in der subjektiven Beurteilung als auch in der Umsetzung im klinischen Alltag. ÄiW in Kanada empfanden das Weiterbildungskonzept als sehr strukturiert. Dort zeigte sich, dass dennoch nur durchschnittlich 8,8 % der wöchentlichen Arbeitszeit in die Weiterbildung investiert wurden [8]. Diese findet in Kanada hauptsächlich an den Universitätskliniken statt. Um die Weiterbildung weiter zu verbessern, erfolgte bereits 2011 die Einbindung eines Simulationstrainings in das Kurrikulum der University of Toronto [9].

In der Schweiz wird zum Teil nur der geringe Anteil von 1,7 % der wöchentlichen Arbeitszeit für die Weiterbildung aufgewendet [8]. Diese wird dort als eher unstrukturiert empfunden [10]. Das Kurrikulum ist durch Zwischenprüfungen geregelt, welche nach 2 Jahren „common trunk“ und anschließend 4 Jahren spezialisiertem Training erfolgen. Hier zeigt sich ein struktureller Unterschied zum Weiterbildungssystem in Deutschland, welches komplett ohne Zwischenprüfungen abläuft [7]. In der Schweiz sind insgesamt vier Weiterbildungskurse verpflichtend und wie in Deutschland ist zum Erlangen des Facharztes die Dokumentation durchgeführter Operationen in einem Logbuch etabliert [10].

**Figure 1:**
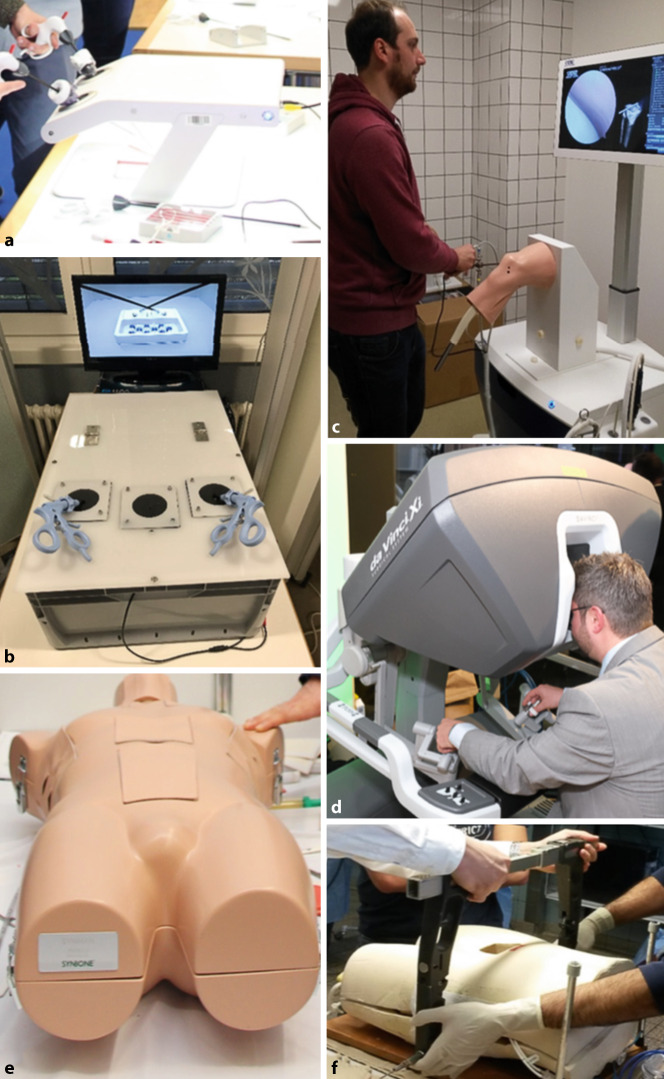

Für das Erlernen praktischer Kompetenzen wie die Augen-Hand-Koordination eignen sich bereits einfache Simulatoren. Erste Schritte können mit diesen bereits außerhalb des Operationssaals eigenständig erlernt und optimiert werden. Einfache Simulatoren – wie beispielsweise Box-Trainer – bestehen in der Regel aus einer Endoskopkamera, einem Monitor und einer Trainingsfläche in unterschiedlicher Form (Würfel, Quader, Halbkugel) mit Portalen zur Nutzung von Kamera und Instrumenten. Innerhalb der Trainingsfläche können Fertigkeiten wie Triangulieren, Instrumentenhandling etc. durch einfache Aufgaben erlernt werden. Box-Trainer bieten hierbei oft nicht den reellen Situs, sind dafür aber kostengünstig in der Beschaffung und Instandhaltung (Abb. 1a, b).

Im Bereich der Orthopädie und Unfallchirurgie kommen außerdem Gelenksimulatoren zum Trainieren arthroskopischer Eingriffe zum Einsatz. Diese sind ähnlich wie die Box-Simulatoren aufgebaut, bieten aber den zusätzlichen Nutzen einer anatomischen Region und somit ein erstes Verständnis des intraartikulären Operationssitus. Der Vorteil solcher anatomischen Simulatoren ist, dass einfache Übungen wie Meniskusresektion oder die Platzierung von Ankersystemen komplikationslos und ohne großen Aufwand trainiert werden können. Zu berücksichtigen ist, dass die „operierten“ anatomischen Strukturen regelmäßig ausgetauscht werden müssen. Offene Operationen lassen sich an offenen Simulatoren, anatomischen Präparaten oder mithilfe von Tiermodellen trainieren [9]. Letztere sind in der praktischen Umsetzung oft logistisch aufwendig und können nicht an jedem Standort angeboten werden [5].

In der Folge des technologischen Fortschritts sind mittlerweile auch Simulatoren mit einer virtuellen Realität (VR) oder augmentierten Realität (AR)/Mixed Reality (MR) für das chirurgische Training verfügbar [11]. Das integrierte haptische Feedback ermöglicht die Durchführung kompletter chirurgischer Eingriffe am Simulator. Ebenfalls offeriert die Nutzung von AR/MR mithilfe einer entsprechenden Brille auf eine reale Oberfläche projizierbare Simulationen (Abb. 1c, d). Hierbei stehen aktuell vor allem technisch gut umsetzbare viszeralchirurgische, arthroskopische und endovaskuläre Operationstechniken im Fokus [11, 12]. Die Hand-Bildschirm-Koordination kann unter Supervision und zeitnahem Feedback trainiert werden. Ebenfalls wird insbesondere unerfahrenen Chirurgen die Möglichkeit gegeben, den gesamten Ablauf einer Operation zu trainieren sowie die Anatomie zu rekapitulieren.

Alaker et al. zeigten in einer Metaanalyse, dass Probanden, die ein VR-Training durchliefen, signifikant bessere Ergebnisse in der Durchführung und dem zeitlichen Ergebnis erzielten als die Vergleichsgruppe ohne Training [13]. Einheitliche Standards in der Bewertung und Analyse konnten im Rahmen der Literaturrecherche der genannten Studie nicht eruiert werden. So weisen sowohl die operativen Eingriffe (Knotentraining bis hin zu kompletten Cholezystektomien) als auch die Parameter der Erfolgskontrolle große Unterschiede auf. Hierdurch wird die Vergleichbarkeit einzelner Studien erschwert [13]. Ein häufig genutzter Parameter ist die Zeit. Diese allein stellt jedoch keinen ausreichend suffizienten Surrogatparameter zur Bewertung der erfolgreichen Durchführung eines chirurgischen Eingriffs dar [13].

Erste Studien zeigten bereits einen Rückgang der Letalität in Zentren, an denen Simulationstraining zum Einsatz kommt [14].

Neben den praktischen operativen Fähigkeiten können durch das simulatorbasierte Training auch weitere Kompetenzen wie interpersonale Skills durch Teamwork, Wissenstransfer und Management komplexer Abläufe sowie chirurgische Notfallbehandlungen (Abb. 1e, f) auf hohem Niveau erlernt werden [15]. Für eine bundesweite Implementierung von Simulationstraining in die Weiterbildung müssen ebenfalls die damit verbundenen Kosten und die bis dato eingeschränkte Zugänglichkeit zu den Geräten Berücksichtigung finden [16].

Das Ziel der vorliegenden Umfragestudie war die Analyse des aktuellen Einsatzes und der Verteilung chirurgischer Simulatoren an deutschen Kliniken. Vergleichbare Erhebungen wurden bereits in Großbritannien durchgeführt [17]. In der hier vorliegenden Studie wird erstmalig ein deutschlandweiter Überblick über die Verteilung und den Einsatz chirurgischer Simulatoren gegeben.

## Methoden

### Studiendesign und Datensammlung

Die hier vorliegende Studie wurde im Jahr 2019 über einen Zeitraum von 6 Monaten (Mai bis Oktober 2019) durchgeführt. Hierzu wurde ein onlinebasierter elektronischer Fragebogen über SurveyMonkey (SurveyMonkey Europe UC, Dublin, Irland) mit insgesamt 19 standardisierten Fragen erstellt. Die Verteilung des Links zum Fragebogen erfolgte an die leitenden chirurgischen Klinikärzte über die E‑Mail-Verteiler der Deutschen Gesellschaft für Chirurgie und den angegliederten 10 Fachgesellschaften. Die Teilnahme an der Umfrage war anonym und freiwillig. Im Sinne des Datenschutzes wurden keine persönlichen Daten (E-Mail, Adresse, Namen etc.) an die Untersucher weitergegeben. Als Einschlusskriterium wurde die Ausweisung als chirurgische Klinik im Bereich des stationären Sektors definiert. Insgesamt wurden 2358 leitende chirurgische Ärzte angeschrieben.

### Fragebogen und Themenkomplexe

Der onlinebasierte Fragebogen setzte sich aus vier Themenkomplexen mit insgesamt 19 standardisierten Fragen zusammen (Tab. 1). Zunächst erfolgte die Zuordnung eines chirurgischen fachspezifischen Bereichs (Allgemein- und Viszeralchirurgie, Gefäßchirurgie, Herzchirurgie, Kinderchirurgie, Mund‑, Kiefer- und Gesichtschirurgie, Neurochirurgie, Orthopädie- und Unfallchirurgie, plastische und ästhetische Chirurgie sowie Thoraxchirurgie).

**Table 1:** 

In welcher Klinikform sind Sie tätig?
Lehrkrankenhaus?
Welchen Träger hat Ihre Klinik?
In welchem Fachbereich sind Sie tätig?
In welchem Bundesland arbeiten Sie?
Halten Sie es für sinnvoll, Simulationstraining zukünftig in die Facharztweiterbildung zu integrieren?
Fall Sie noch keinen Simulator an Ihrer Klinik haben – planen Sie die Neubeschaffung eines solchen Gerätes?
Gibt es einen chirurgischen Simulator an Ihrer Klinik? (Keine Reanimationsimulatoren)
Welches Gerät ist an Ihrer Klinik vorhanden? (Mehrfachantwort möglich)
Ist das Simulatortraining aktiv in die Weiterbildung an Ihrer Klinik integriert?
Gibt es Schulungen zur Anwendung des Geräts?
Gibt es einen klinikinternen Rotationsplan zur Nutzung des Geräts?
Ist das Gerät für ärztliche MitarbeiterInnen unlimitiert zugänglich?
Können Studierende den Simulator nutzen?
Gibt es eine Kooperation mit der Anatomie mit der Möglichkeit, Zugangswege am Präparat zu trainieren?
Wie hoch ist die Nutzungszeit pro Assistenzarzt/-ärztin?
Wer nutzt das Simulatortraining am meisten?

Die weiteren abgefragten Inhalte bildeten die Themenkomplexe Klinikart, Nutzungsverhalten, geographische Verteilung und subjektive Einschätzung der Bedeutung des Simulatortrainings für die chirurgische Weiterbildung ab.

Drei Fragen bezüglich der Klinikart wiesen die Trägerschaft, die Form und eine vorhandene Eigenschaft als Lehrkrankenhaus aus. Der Themenkomplex „Bedeutung“ enthielt zwei Fragen mit subjektiver Bewertung zur aktuellen und zukünftig geplanten Integration von Simulatoren in die chirurgische Weiterbildung.

Von besonderem Interesse für die Analyse war das Nutzungsverhalten. Dieses wurde über insgesamt 11 Fragen für die jeweiligen Standorte analysiert. Das Anwendungsverhalten wurde auf einer numerischen Skala von 1 bis 5 nach Häufigkeit bewertet.

### Statistik

Die statistische Auswertung erfolgte als deskriptive Statistik mittels Excel (Microsoft, Redmond, WA, USA) und Sigmaplot 13 (Systat, San Jose, CA, USA).

## Ergebnisse

**Figure 2:**
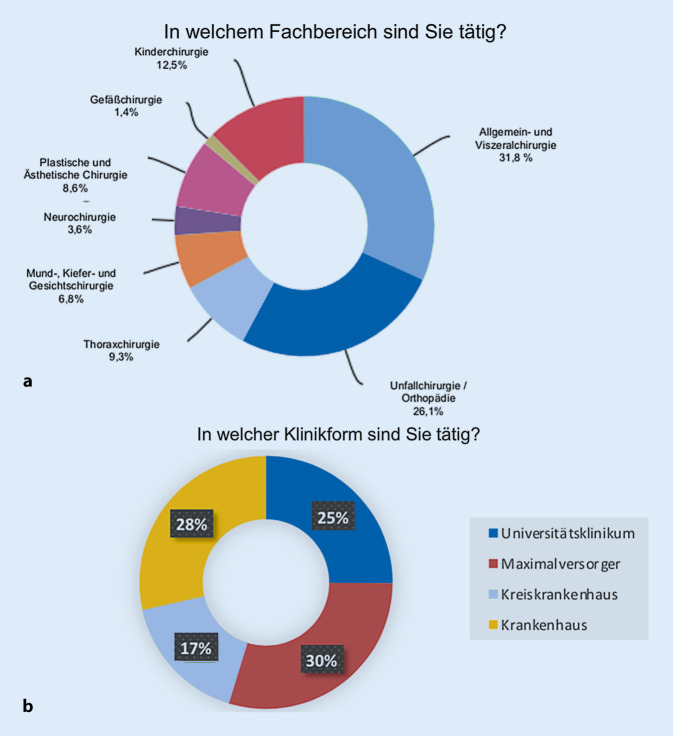

Die Umfragestudie wurde von 283 Teilnehmern beantwortet. Nach Ausschluss doppelt eingegeben Daten oder unvollständiger Datensätzen verblieben 267 vollständige Datensätze. Insgesamt ergab sich eine Rücklaufquote von 12 %. Die Fächer Allgemein- und Viszeralchirurgie sowie Orthopädie und Unfallchirurgie machten einen Gesamtanteil von 57,0 % an der vorliegenden Studie aus (Abb. 2a).

Es zeigte sich eine Verteilung der teilnehmenden Kliniken mit 26,2 % Universitätskliniken, 30,4 % Maximalversorger, 16,3 % Kreiskrankenhäuser und 26,2 % Krankenhäuser (Abb. 2b). 84,0 % der Teilnehmer waren an Lehrkrankenhäusern tätig. Die Krankenhausträgerschaft lag für öffentliche Träger bei 54,0 %, für kirchliche bei 28,1 % und private bei 17,9 %.

Es gab Beantwortungen aus allen Bundesländern, wobei Nordrhein-Westfalen (23,5 %) und Bayern (17,3 %) die höchsten Beantwortungsquoten aufwiesen.

**Figure 3:**
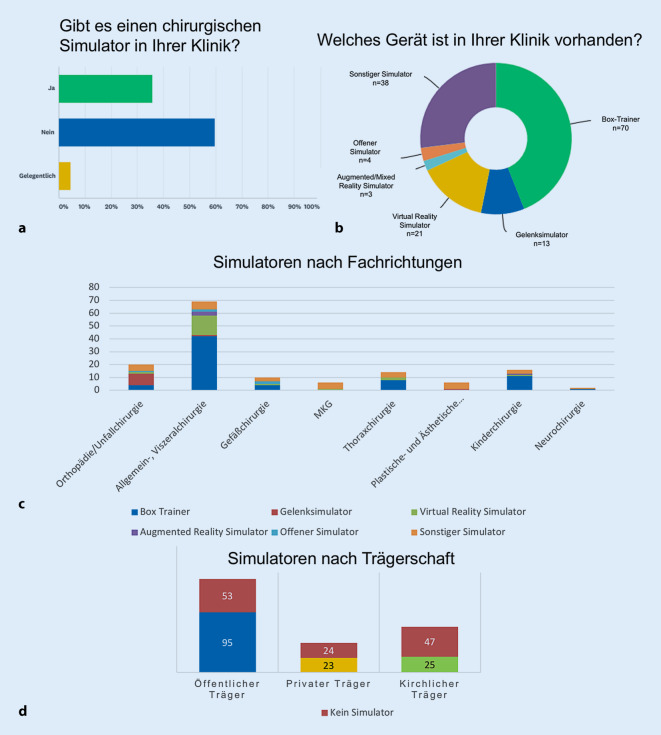

Das Vorhandensein chirurgischer Simulatoren wurden von 35,0 % (*n* = 143) der Teilnehmer bestätigt (Abb. 3a). Einzelne Kliniken besitzen zum Teil mehrere Simulatoren. Ein Teil der Kliniken (4,6 %) kann nur gelegentlich auf Simulatoren zurückgreifen. 60 % der Kliniken hatten keinen Simulator.

Das am häufigsten vorhandene Simulatormodell ist der Box-Trainer (*n* = 70). Als weitere Modelle wurden sonstige Simulatoren wie Eigenbauten (*n* = 32), Virtual-Reality-Simulatoren (*n* = 21), Gelenksimulatoren (*n* = 11), offene Simulatoren (*n* = 5) und Augmented‑/Mixed-Reality-Simulatoren (*n* = 3) angegeben (Abb. 3b).

Zwischen den Fachrichtungen zeigte sich, dass endoskopisch/arthroskopisch operative Fächer auch vermehrt chirurgische Simulatoren besitzen (Abb. 3c). So kommen die meisten Box-Trainer in der Allgemein- und Viszeralchirurgie (*n* = 42) sowie der Kinderchirurgie (*n* = 11) und Thoraxchirurgie (*n* = 8) zum Einsatz (Gefäßchirurgie *n* = 4, Orthopädie/Unfallchirurgie *n* = 4, Neurochirurgie *n* = 1). Gelenksimulatoren (*n* = 9) kommen fast ausschließlich in der Orthopädie/Unfallchirurgie zum Einsatz. VR-Simulatoren sind überwiegend in der Allgemein- und Viszeralchirurgie (*n* = 15) vorhanden. In den Fachdisziplinen Thoraxchirurgie (*n* = 2), Gefäßchirurgie (*n* = 1), Kinderchirurgie (*n* = 1), Mund‑, Kiefer- und Gesichtschirurgie (*n* = 1) und Orthopädie/Unfallchirurgie (*n* = 1) waren vereinzelt VR-Simulatoren vorhanden. AR‑/MR-Simulatoren werden bis dato in der Allgemein- und Viszeralchirurgie (*n* = 3) und der Kinderchirurgie (*n* = 1) genutzt. Offene Simulatoren werden in der Allgemein- und Viszeralchirurgie (*n* = 2), der Gefäßchirurgie (*n* = 2) und der Orthopädie/Unfallchirurgie (*n* = 1) eingesetzt. Sonstige Simulatoren kommen in allen Fächern zum Einsatz (Allgemein- und Viszeralchirurgie *n* = 6, Orthopädie/Unfallchirurgie *n* = 5, Gefäßchirurgie *n* = 3, Mund‑, Kiefer- und Gesichtschirurgie *n* = 5, plastische und ästhetische Chirurgie *n* = 5, Thoraxchirurgie *n* = 4, Kinderchirurgie *n* = 3 und Neurochirurgie *n* = 1).

Kliniken öffentlicher Trägerschaft verfügen insgesamt über 95 Simulatoren, private Klinikträger über 23 und kirchliche Klinikträger über 25 (Abb. 3d).

**Figure 4:**
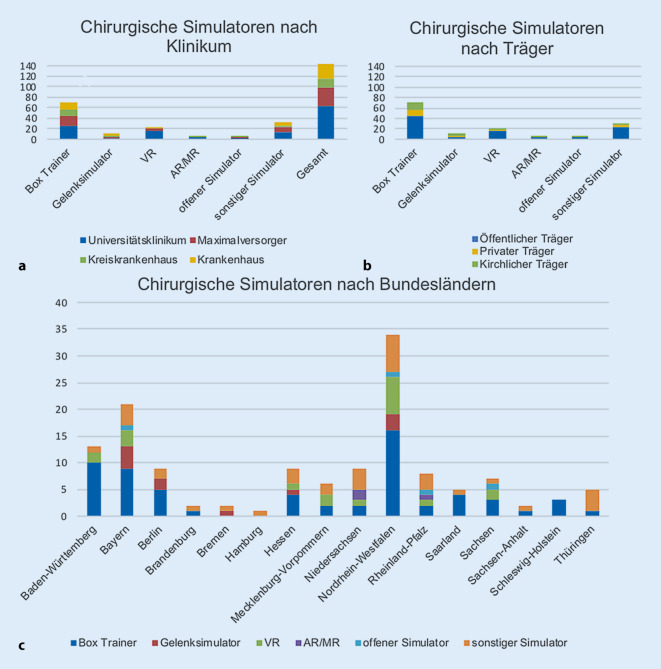

In Bezug auf die Klinikart (Universitätsklinikum, Maximalversorger, Kreiskrankenhaus, Krankenhaus; Abb. 4a) zeigten sich Box-Trainer und Gelenksimulatoren an allen Kliniken, Virtual-Reality- (*n* = 17) und AR/MR-Simulatoren (*n* = 3) befanden sich fast ausschließlich an Universitätskliniken. VR-Simulatoren waren außerdem an 3 Kliniken der Maximalversorgung und in einem Krankenhaus vorhanden. Offene Simulatoren waren insgesamt nur gering verfügbar (Universitätskliniken *n* = 2, Maximalversorgen *n* = 2 und Kreiskrankenhaus *n* = 1). Sonstige Simulatoren waren an Universitätskliniken (*n* = 14), Maximalversorgern (*n* = 9), Kreiskrankenhäusern (*n* = 2) und Krankenhäusern (*n* = 7) vorhanden (Abb. 4a). Es zeigte sich, dass Box-Trainer unabhängig von der Trägerschaft vorhanden sind. VR-Simulatoren und AR-/MR-Simulatoren waren nur an Kliniken mit öffentlicher Trägerschaft vorhanden (Abb. 4b).

Die meisten Simulatoren waren in den Bundesländern Baden-Württemberg, Bayern und Nordrhein-Westfalen vorhanden (Abb. 4c). In den Bundesländern Berlin, Brandenburg, Bremen, Hamburg, Hessen, Saarland, Sachsen-Anhalt, Schleswig-Holstein und Thüringen waren keine VR- oder AR-Simulatoren vorhanden.

Bezüglich des Anwendungsverhaltens zeigte sich ein überwiegender Einsatz der Simulatoren durch Studierende, gefolgt von Ärzten in Weiterbildung, Fachärzten, Oberärzten und Chefärzten. Die Zeit am Simulator für ÄiW war zu 53,2 % unbekannt. Von den bekannten Nutzern wurde angegeben, dass der Simulator meist nur einmal im Monat verwendet wurde.

Insgesamt 46,0 % der Umfrageteilnehmer bestätigten die Möglichkeit zur uneingeschränkten Nutzung für Studierende. Ungefähr die Hälfte aller ärztlichen Mitarbeiter (50,3 %) hatte unlimitierten Zugang zu den vorhandenen Simulatoren.

Eine Integration des Simulatortrainings in die chirurgische Facharztweiterbildung bestand für die überwiegende Mehrheit (81,8 %) bisher nicht. 94,5 % hielten dies zukünftig jedoch für sinnvoll. 21,1 % der Teilnehmer ohne Simulator an der eigenen Klinik planten die Anschaffung eines neuen Gerätes.

An den entsprechenden Kliniken wurden zu 34,1 % Schulungen zur Anwendung des Geräts durchgeführt. An 19,8 % der Kliniken gibt es gelegentliche Schulungen und an 46,2 % keine. Darüber hinaus fand jedoch kein strukturierter Einsatz der Simulatoren statt. In 77,7 % der Fälle existierte kein Rotationsplan für die Nutzung des Simulators.

Die Begleitung des Simulatortrainings durch einen Mentor bestand in 16,5 % der Fälle. Die Möglichkeit des chirurgischen Kompetenzerwerbs an Humanpräparaten in Kooperation mit einer Anatomie war in 14,0 % möglich.

## Diskussion

Reglementierungen durch das Arbeitszeitgesetz, die Arbeitsverdichtung im klinischen Alltag sowie der Fachkräftemangel in den chirurgischen Fachdisziplinen erfordern die Entwicklung innovativer Strategien, um den Herausforderungen einer qualitativ hochwertigen Weiterbildung gerecht zu werden [1, 3, 18].

Durch ökonomische Erfordernisse und Regulationen durch das europäische Arbeitszeitgesetz kann außerdem angenommen werden, dass insbesondere technisch anspruchsvolle operative Eingriffe des Weiterbildungskatalogs im klinischen Alltag zunehmend schwieriger umzusetzen sind. Eine Möglichkeit dem entgegenzuwirken wäre hier eine Simulation dieser Eingriffe, um die erworbenen Kompetenzen im operativen Setting zielgerichteter umsetzen zu können. Dabei bietet das Simulationstraining die Möglichkeit, verschiedenste Interventionen sowie auch Prozessabläufe zu trainieren [10, 19, 20] Miller et al. zeigten im Jahr 2012 die Kompetenzentwicklung in den Bereichen Teamwork und Kommunikation [20]. Ebenso sind die Vermittlung von Wissen, das Training praktischer Fähigkeiten und das Erschließen komplexer Behandlungsabläufe beschrieben [5, 21]. In einem systematischen Review konnte der erfolgreiche Transfer von simulationsbasiertem Kompetenzerwerb in das operative Setting gezeigt werden [5].

Die vorliegende Studie gibt erstmalig einen Überblick über den aktuellen Stand der Verteilung und Anwendung chirurgischer Simulatoren in Deutschland. Die Datensammlung basiert auf einer Onlineumfrage. Durch den versendeten Teilnahmelink konnten verschiedene Kliniken und chirurgische Fachdisziplinen erreicht werden. Die Rücklaufquote entsprach ähnlich konzipierten Umfragestudien [22, 23]. Hierbei repräsentiert die Umfrage insgesamt 13,7 % aller chirurgischen Fachabteilungen in deutschen Kliniken. Limitationen ergaben sich durch die eingeschränkte Erreichbarkeit von Klinikleitern z. B. aufgrund einer fehlenden Mitgliedschaft in den deutschen Fachgesellschaften.

Der überwiegende Teil der Studienteilnehmer repräsentiert die Situation an Lehrkrankenhäusern und Universitätskliniken. Die vorliegenden Ergebnisse zeigen den Einsatz simulationsbasierten Trainings in allen chirurgischen Fachdisziplinen. Hiernach sind Simulatoren in der Allgemein- und Viszeralchirurgie sowie in der Orthopädie und Unfallchirurgie bis dato am weitesten verbreitet. Entsprechend besteht hier das aktuell größte Angebot auf dem Markt, mit Fokus auf Laparoskopie- und Arthroskopietrainern. Die aktuelle Studienlage zeigt, dass vorrangig Berufsanfänger den höchsten Lerneffekt am Simulator erzielen können [24, 25]. Betrachtet man die Ergebnisse der vorliegenden Studie, zeigt sich, dass Studierenden und ÄiW die häufigste Nutzung der Geräte zugeschrieben wird. Eine genaue Nutzungszeit wird in der Regel nicht erhoben, sodass der Einfluss der Simulatoren in den Kliniken im Hinblick auf dezidierte Nutzungszeiten nicht weitergehend untersucht werden konnte. Es ist anzunehmen, dass eine standardisierte Dokumentation von Nutzungsverhalten, Nutzungsdauer und Effizienz der Trainingskonzepte die Argumentation zur Finanzierung der zum Teil sehr kostenintensiven Geräte gegenüber den Kostenträgern unterstützen würde. Sowohl in der Literatur als auch in unseren Ergebnissen zeigt sich, dass standardisierte Schulungen und strukturierte Trainingszeiten im klinischen Alltag die Ausnahme darstellen [26].

In der Luftfahrt und im Militärwesen ist das Trainieren unvorhersehbarer Ereignisse und Krisensituationen im simulationsbezogenen Setting Routine und Voraussetzung für die Tätigkeit [20]. Durch ein detailliertes Feedback können Kompetenzdefizite aufgezeigt und situationsadaptiert trainiert werden. Die individuell erforderliche Zeit, um den gewünschten Trainingseffekt zu erreichen, sollte in die Routineabläufe der klinischen Versorgung integriert werden [9]. Hierzu ist ein freier Zugang zum Gerät notwendig, was in der genannten Studie nur in der Hälfte der Fälle möglich war. Um Schäden am Gerät durch Informationsdefizite zu vermeiden, sollte zu Beginn eine Instruktion durch einen fachkundigen Mentor erfolgen. Es kann außerdem zu einem größeren Lernerfolg führen, wenn ein strukturiertes Feedback von Mentoren gegeben wird [26]. Um den gewünschten Trainingseffekt zu erreichen, ist ein regelmäßiges und umfangreiches Training notwendig [27]. Die Erstellung von Rotationsplänen könnte eine geeignete Ergänzung darstellen. Analog zu den Operationszahlen sollte ebenfalls eine zielorientierte Kommunikation und Dokumentation von Trainingsinhalten im Rahmen von Feedbackgesprächen kommuniziert und dokumentiert werden.

Rosen et al. zeigten, dass durch Simulationstraining die Operationszeit verkürzt und die postoperative Letalität gesenkt werden kann [28]. 90,0 % der leitenden Ärzte wünschten sich in der Studie die Integration des Simulationstrainings in die Facharztweiterbildung [29]. Etablierte kurrikulare Konzepte für die verschiedenen chirurgischen Fachdisziplinen gibt es bereits in Großbritannien [15] oder in Dänemark, wo 2015 ein chirurgisches Pflichtkurrikulum basierend auf Simulationstraining für alle Ärzte vor Beginn der Weiterbildung etabliert wurde [30]. In der Gefäßchirurgie gibt es in Dänemark beispielsweise einen zweitägigen Kurs über Gefäßanastomosen. Eine retrospektive Auswertung über 8 Jahre zeigte eine Verbesserung der Leistungstests vom 1. auf den 2. Tag bei 83 Teilnehmern [30]. Eine Relation zwischen der klinischen Erfahrung und dem ersten Leistungstest konnte jedoch nicht festgestellt werden. In einer Übersichtsarbeit von Ott et al. wurden die Ergebnisse dahingehend interpretiert, dass die rein klinische Weiterbildung ohne Simulationstraining in Dänemark zum Teil Defizite in Bezug auf die technischen Fertigkeiten der Ärzte in Weiterbildung aufweist [31].

Ein kurrikulares Setting sollte angestrebt werden, um die Vorteile des Simulationstrainings im Sinne einer sicherheitsorientierten und effizienten Patientenversorgung nutzbar zu machen. Entsprechend den Ergebnissen unserer Umfragestudie sind die Anzahl und der Zugang zu chirurgischen Simulatoren limitiert. Nur 35 % der in dieser Studie befragten deutschen Kliniken verfügt überhaupt über einen chirurgischen Simulator. Insbesondere kostenintensive Simulationssysteme wie VR-Simulatoren stehen fast ausschließlich den Universitätskliniken zur Verfügung. Ein entscheidender Punkt bildet somit neben der Größe des Klinikums auch die Trägerschaft. Hierbei zeigte sich, dass ca. 66 % der hier befragten Kliniken in öffentlicher Trägerschaft einen chirurgischen Simulator besitzen. Private Krankenhausträger verfügen zu 50 % und die kirchlichen Träger lediglich zu 33 % über Simulatoren. Auch zeigten sich regional starke Unterschiede. Die neuen Bundesländer weisen in der Summe die gleiche Anzahl von Simulatoren wie Bayern auf. Um weiterhin eine bundesweit gleichwertige Patientenversorgung und Weiterbildung gewährleisten zu können, sollten neue Konzepte des Ausbaus geeigneter Strukturen auf Bundesebene erarbeitet und implementiert werden. Hieraus lässt sich schlussfolgern, dass an Kliniken ohne entsprechende finanzielle Investition die Weiterbildung in einem vorgegebenen kurrikularen Setting nur eingeschränkt oder gar nicht erfolgen könnte. Somit stellt, neben der Frage der Einbindung in das chirurgische Kurrikulum, auch die Finanzierung solcher Systeme einen essenziellen Diskussionspunkt dar. Eine Möglichkeit wäre die zentrale Nutzung, ebenfalls nach einem festen Kurrikulum. Dadurch könnte in regionalen Simulationszentren langfristig die Nutzung und Auslastung der Geräte erhöht und gleichzeitig die Finanzierung erleichtert werden.

Ein solches Konzept wurde bereits in den USA durch das American College of Surgeons als Surgery Resident Skills Curriculum etabliert (FACS). Hierbei durchlaufen ÄIW drei Stufen der Simulation im Rahmen ihrer Weiterbildung. Die ÄiW können dadurch praktische, aber auch teambezogene Fertigkeiten erlernen [29].

Zu berücksichtigen ist ebenfalls die Notwendigkeit einer effizienten Nutzung von Operationskapazitäten als Hochkostenbereich einer Klinik [32]. Hier trägt simulationsbasiertes Training eindeutig zur Entlastung des Operationsmanagements bei. Ein weiterer Vorteil des Simulationstrainings besteht in dem Erlernen von Kompetenzen, deren Training im klinischen Setting aufgrund des spezifischen Patientenklientels (z. B. Kinder, Hochrisikopatienten) eine besondere Herausforderung darstellt. Weiterhin ist zu berücksichtigen, dass die gesundheitsökonomische Bedeutung der Weiterbildung und die damit einhergehenden Kosten bis dato nicht durch das Diagnosis-related-Groups(DRG)-System abgebildet sind. Es ist jedoch anzunehmen, dass das Erlernen praktischer Fähigkeiten durch supervisiertes Simulatortraining mit einer modifizierten Verfügung zeitlicher und personeller Ressourcen verbunden ist. Dies sollte im Sinne der Qualitätssicherung bei der Entwicklung kurrikularer Weiterbildungskonzepte Berücksichtigung finden. Finanzielle Investitionen sollten ferner primär das Ziel der Kompetenzentwicklung der Chirurgen und damit die Qualitätssicherung der Patientenversorgung verfolgen.

Simulatoren sind in verschiedenen Anwendungsdimensionen bereits am Markt erhältlich und deren Effektivität unter Supervision ist hierbei in der Literatur beschrieben [26]. Entsprechend sollten die bereits vorhandenen Simulatoren künftig durch strukturierte Kurrikula in die Weiterbildungskonzepte der Kliniken integriert werden und die Verfügbarkeit flächendeckend erhöht werden, um eine qualitativ hochwertige Weiterbildung auch weiterhin gewährleisten zu können. Des Weiteren sollte Simulationstraining in die Weiterbildungsordnungen aufgenommen werden, hierbei können bereits etablierte internationale Konzepte als Blaupause dienen und die verfügbaren Innovationen genutzt werden.

## Schlussfolgerung

Chirurgische Kompetenzen können in der Aus‑, Weiter- und Fortbildung durch simulationsbasiertes Training außerhalb des Operationssaals selbstständig oder mit Anleitung ohne Gefährdung der Patientensicherheit erlernt werden. Simulationsbasiertes Training ist in anderen Branchen – wie beispielsweise der Luftfahrt – seit langem etabliert. Die Ergebnisse der vorliegenden Studie zeigen, dass 35 % der befragten chirurgischen Kliniken in Deutschland über ein Simulatorsystem verfügen. Bei vorhandenen Simulatoren wird das tatsächliche Nutzungsverhalten kaum dokumentiert. Eine modifizierte und strukturierte Integration von Simulationstraining in die chirurgische Aus‑, Weiter- und Fortbildung bildet eine zukunftsorientierte Möglichkeit, die sicherungstechnischen, didaktischen und ökonomischen Vorteile für alle chirurgischen Fachdisziplinen nutzbar zu machen.

## Limitationen

Es handelt sich bei dieser Studie um eine Umfragestudie, welche von der Rücklaufquote der Befragten abhängig ist und nur einen repräsentativen Überblick geben kann, da nicht alle deutschen chirurgischen Kliniken befragt wurden. Somit kann die genaue Anzahl verfügbarer chirurgischer Simulatoren in Deutschland nur abgeschätzt werden.
